# Presentation of Lemierre’s Syndrome Secondary to Klebsiella pneumoniae-Caused Neck Abscess Following an Odontogenic Infection

**DOI:** 10.7759/cureus.87882

**Published:** 2025-07-14

**Authors:** Jing Wen Wong, Jia Yee Pwi, Siew Ping Lau, Chee Yik Chang

**Affiliations:** 1 Internal Medicine, Hospital Sultanah Aminah, Johor Bahru, MYS; 2 Infectious Diseases, Hospital Sultanah Aminah, Johor Bahru, MYS

**Keywords:** ijv thrombosis, klebsiella pneumoniae, lemierre's, lemierre's syndrome, odontogenic, venous thrombophlebitis (ijv), venous thrombosis (ijv)

## Abstract

Lemierre’s syndrome is a rare but potentially fatal complication of oropharyngeal or odontogenic infections, typically caused by *Fusobacterium necrophorum*. In rare instances, *Klebsiella pneumoniae *(*K. pneumoniae*) has been identified as the causative organism. It is characterized by septic thrombophlebitis of the internal jugular vein (IJV) and may result in septic emboli to distant organs. We describe the case of a 54-year-old woman with poorly controlled diabetes mellitus who presented with a deep neck space infection following a tooth extraction. Imaging revealed multiple deep neck abscesses, mandibular osteomyelitis, and thrombosis of the right IJV. Surgical drainage was performed, and cultures revealed *K. pneumoniae*. She recovered well after a six-week course of antibiotics and anticoagulation.

## Introduction

Odontogenic infections are a common cause of deep neck space infections, typically originating from dental caries or post-extraction complications. While most cases remain localized, spread to adjacent fascial planes can lead to life-threatening complications, particularly in immunocompromised individuals such as those with poorly controlled diabetes mellitus [1]. Among the rare but serious complications is internal jugular vein (IJV) thrombosis, a hallmark of Lemierre’s syndrome, classically associated with *Fusobacterium necrophorum* but increasingly recognized with other pathogens: a condition termed Lemierre-like syndrome [2]. *Klebsiella pneumoniae* (*K. pneumoniae*) has rarely been implicated in such presentations [3].

We report a case of a diabetic woman who developed extensive deep neck space abscesses, mandibular osteomyelitis, and right IJV thrombosis following a dental extraction, with *K. pneumoniae* identified as the causative organism. This case highlights the importance of prompt diagnosis, multidisciplinary management, and awareness of uncommon complications arising from odontogenic infections.

## Case presentation

A 54-year-old Malay woman with a history of type 2 diabetes mellitus, suboptimally controlled on oral metformin 1 g twice daily, presented with a five-day history of progressively worsening right submandibular pain and swelling. The swelling extended superiorly towards the right preauricular region and was associated with odynophagia and dysphagia. Two months prior, she had undergone extraction of the right lower first molar (tooth 46), complicated by transient right submandibular swelling that resolved spontaneously without antibiotic treatment.

On examination, there was a diffuse, firm, warm, and tender swelling with overlying erythema over the right infra-auricular region extending to level II submandibular and submental areas. Purulent discharge was observed from the previous dental extraction site, where irrigation was performed and extraction of tooth 47 was done. The remainder of the systemic examination was unremarkable.

Laboratory investigations revealed a mildly elevated white blood cell count of 12.4 × 10⁹/L (normal range: 4.0-10.0 × 10⁹/L) and markedly raised C-reactive protein (CRP) of 189.3 mg/L (normal: <5 mg/L). Renal and liver function tests were within normal limits. Capillary blood glucose levels ranged from 15 to 17 mmol/L (normal fasting glucose: 3.9-5.5 mmol/L).

**Figure 1:**
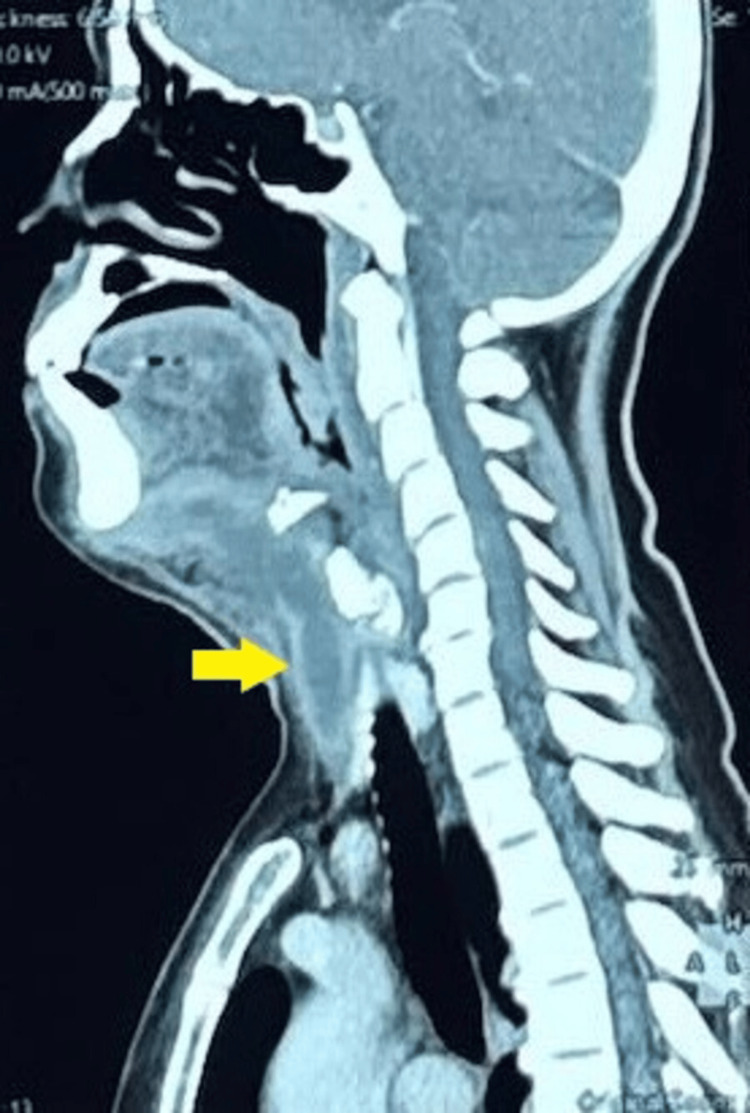
CT of the neck showing multiloculated communicating right cervical collections in the right parapharyngeal and right submandibular spaces, indicating abscess formation (indicated by arrow)

**Figure 2:**
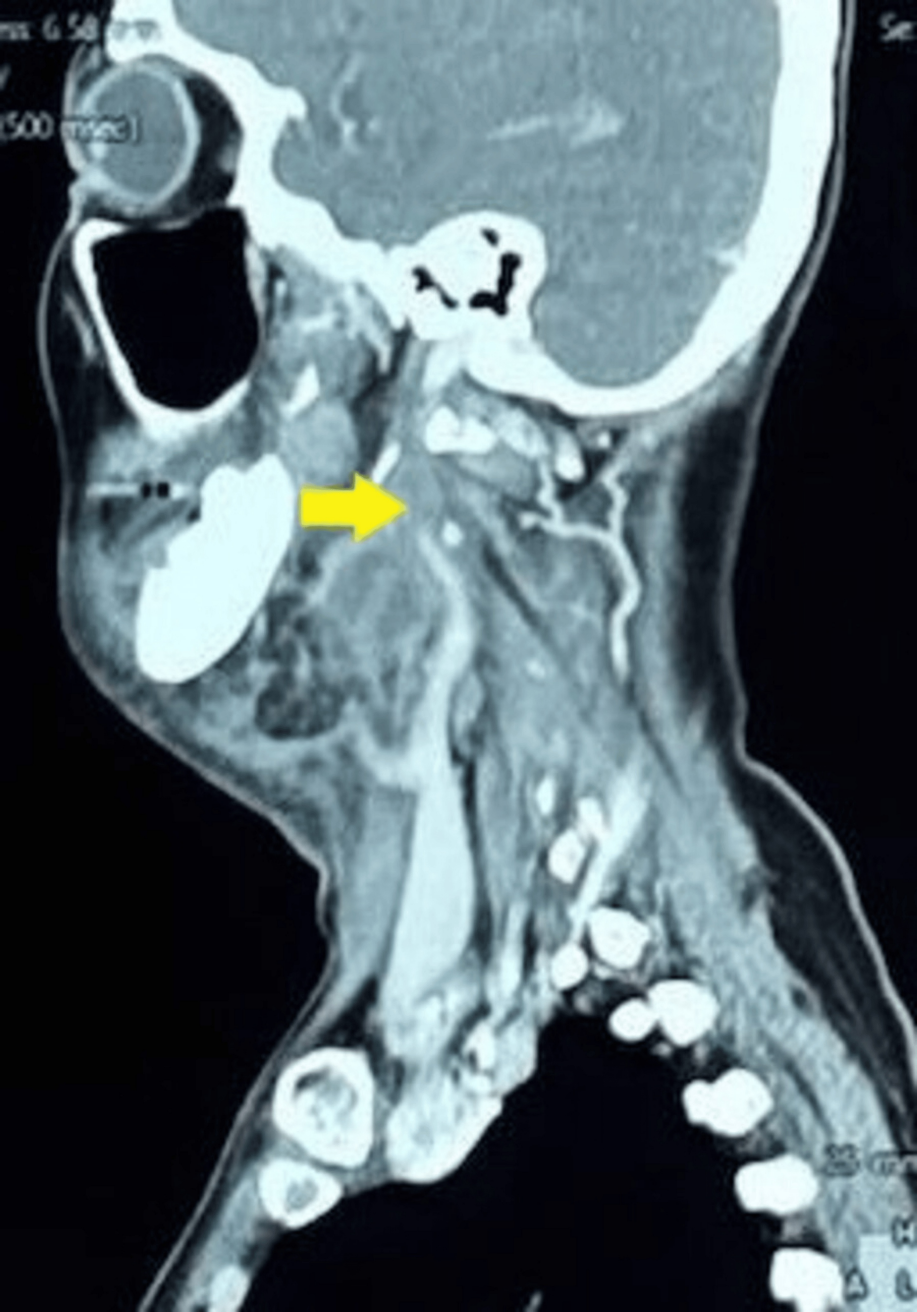
CT neck shows a rim-enhancing collection in the right parapharyngeal and submandibular spaces with surrounding inflammation, causing compression and poor visualization of the right IJV, raising suspicion for thrombosis (indicated by arrow) IJV: Internal jugular vein

The initial clinical impression was right submandibular cellulitis of odontogenic origin with a possible abscess. Empirical intravenous amoxicillin-clavulanic acid (1.2 g every eight hours) was initiated, and a basal-bolus insulin regimen was started for glycemic control. Contrast-enhanced CT of the neck revealed right parapharyngeal and submandibular multiloculated, rim-enhancing collections, right mandibular osteomyelitis, and thrombosis of the right IJV (Figures 1, 2). 

The patient underwent incision and drainage of the abscess. Intraoperatively, erythematous and inflamed right submental and submandibular skin with thickened platysma was noted. Three distinct abscess locules were identified: one extending from the submental region to the contralateral side, superiorly to the mandibular ramus and inferiorly to the hyoid bone; another deep to the strap muscles from below the cricoid cartilage to the hyoid; and a third located superior to the angle of the mandible medial to the carotid artery. Approximately 40 mL of pus was drained.

Cultures of pus and tissue grew *K. pneumoniae*, sensitive to amoxicillin-clavulanic acid, ampicillin-sulbactam, and piperacillin-tazobactam. Acid-fast bacilli staining and mycobacterial cultures were negative. Histopathological examination of the abscess wall revealed infiltration of neutrophils with scattered lymphocytes, plasma cells, and histiocytes, findings are in keeping with abscesses. Intravenous amoxicillin-clavulanic acid was continued according to susceptibility results. Due to confirmed right IJV thrombosis, subcutaneous enoxaparin 60 mg twice daily (1 mg/kg) was initiated.

The patient’s clinical condition improved with no bleeding complications. At discharge, the white cell count reduced to 12.3 × 10⁹/L and CRP decreased significantly to 6.2 mg/L. After two weeks of intravenous antibiotics, therapy was transitioned to oral amoxicillin-clavulanic acid to complete a planned six-week course. Enoxaparin was switched to oral rivaroxaban (15 mg twice daily for two weeks, then 20 mg once daily for four weeks).

Follow-up CT performed at six weeks demonstrated resolution of the abscess and recanalization of the right IJV. Antibiotics and anticoagulation were subsequently discontinued. The patient continued to be seen at the primary care clinic for diabetes mellitus management.

## Discussion

Lemierre’s syndrome is an uncommon but potentially life-threatening sequela of oropharyngeal infection, characterized by septic thrombophlebitis of the IJV and hematogenous dissemination of septic emboli, most frequently to the pulmonary vasculature [2,3]. Although classically described in young, immunocompetent individuals, an increasing number of cases have been reported in patients with underlying medical conditions, including poorly controlled diabetes mellitus, immunosuppressive conditions, and chronic or recurrent odontogenic or pharyngeal infections [3,4]. 

*Fusobacterium necrophorum*, a Gram-negative anaerobic bacillus that forms part of the normal upper respiratory tract flora, remains the most common causative pathogen. However, increasing reports have documented polymicrobial infections and the involvement of other organisms, including Gram-positive cocci and Enterobacteriaceae such as *K. pneumoniae*, particularly in diabetic patients [3,5-7]. These non-classical pathogens have led to the broader term Lemierre-like syndrome being applied in such cases.

Pathophysiologically, the infection begins with localized inflammation of the tonsillar, peritonsillar, or dental tissues, which then spreads to the parapharyngeal space. The proximity of the IJV allows for direct extension, resulting in thrombophlebitis. From here, septic emboli may disseminate to distant organs, especially the lungs, potentially leading to respiratory distress or septic shock, both associated with high morbidity and mortality [8].

Empirical intravenous antibiotics remain the cornerstone of treatment, with regimens typically covering anaerobic, Gram-positive, and Gram-negative organisms. The duration of therapy often extends to four-six weeks, depending on the presence of complications such as osteomyelitis or septic emboli [3,9]. In this case, *K. pneumoniae* was isolated from the pus culture and was susceptible to amoxicillin-clavulanate, which was continued as definitive therapy with good clinical response.

Surgical intervention, such as incision and drainage, is warranted in cases of abscess formation, particularly when there is failure of medical management or the presence of large, loculated collections [3]. Our patient required operative drainage of multiple abscess compartments, which, in combination with antimicrobial and anticoagulant therapy, led to a successful outcome.

The role of anticoagulation in Lemierre’s syndrome remains controversial. While not routinely recommended in uncomplicated cases, anticoagulation is often considered when there is an extension of thrombus, lack of improvement on antibiotics, or risk of cavernous sinus involvement [10,11]. In our case, anticoagulation was initiated with low molecular weight heparin and later transitioned to direct oral anticoagulant therapy, with eventual recanalization of the IJV noted on follow-up imaging.

## Conclusions

This case illustrates a rare presentation of Lemierre’s syndrome caused by *K. pneumoniae* following an odontogenic infection in a patient with poorly controlled diabetes mellitus. Lemierre-like syndrome should be considered in patients with deep neck infections and IJV thrombosis. Early recognition, timely imaging, surgical drainage, targeted antibiotics, and appropriate anticoagulation are crucial for optimal outcomes.
